# *KRAS* Protein Expression in Oral Squamous Cell Carcinoma: A Potential Marker for Progression and Prognosis

**DOI:** 10.30699/IJP.2022.550727.2856

**Published:** 2022-09-10

**Authors:** Hala M. El Hanbuli, Mostafa A. Abou Sarie

**Affiliations:** *Department of Pathology, Faculty of Medicine, Fayoum University, Egypt *

**Keywords:** Bcl2, Cyclin D1, Ki-67, *KRAS*, Oral squamous cell carcinoma

## Abstract

**Background & Objective:**

Emerging evidence suggests that *KRAS* could play an important role in squamous cell carcinoma; however, its role in oral squamous cell carcinoma (OSCC) is largely unknown. The aim of the current study was to investigate the expression of *KRAS*, Ki-67, Cyclin D1, and Bcl2 in OSCC and their association with clinicopathological features.

**Methods:**

Forty paraffin blocks of retrospective histologically diagnosed cases of OSCC and 20 blocks of oral leukoplakia with epithelial dysplasia were obtained from two hospitals between 2018 and 2021. The paraffin-embedded tissue was analyzed for the expression of *KRAS* for oral epithelial dysplasia and OSCC, and ki-67, Cyclin D1, and *bcl2* were analyzed only for OSCC. The results were correlated with each other and with different clinicopathological features and were statistically analyzed.

**Results:**

*KRAS* expression was significantly associated with histological tumor grade, tumor extent, presence of nodal and distant metastasis, pathological stage, and the presence of lymphovascular invasion (*P*=<0.001, 0.001, 0.001, 0.009, <0.001, and <0.001, respectively). The *KRAS* expression was positively correlated with the histological grade, tumor extent, nodal status, and the pathological stage (r=0.712, 0.649, 0.646, and 0.865, respectively). A positive correlation was also found with the expression of Bcl2, Cyclin D1, and Ki-67 (r=0.81, 0.723, and 0.698, respectively). The *KRAS* expression in oral epithelial dysplasia was significantly lower than that in OSCC (*P*=0.003).

**Conclusion:**

*KRAS* may be a potential prognostic marker for OSCC and may play a role in its progression.

## Introduction

Oral cancer includes the malignant neoplasm of the oral cavity from the lips to the palatoglossal arch. It is considered as a worldwide health problem that is characterized by its high mortality and morbidity (1). About 90–95% of oral cancer cases are oral squamous cell carcinoma (OSCC) (2). The most common malignant tumor is represented in the oral cavity with a high incidence of nodal metastasis and recurrences (3). 

The main risk factors associated with OSCC include smoking, alcohol consumption, infection with high-risk human papillomavirus, and a diet low in fruits and vegetables (4,5). OSCC more commonly affects men above 40 years (6). Any site in the oral cavity can be affected, however, the tongue and the floor of the mouth are the most frequently affected sites (7). 

Resection and reconstruction are the commonest treatments of OSCC (1). Despite recent advances in treatment modalities (surgery, radiotherapy, chemotherapy), OSCC still has a poor prognosis, with a recurrence rate of about 30% in the cases. In addition, these treatments have been associated with significant side effects (8).

The principal causes of OSCC-related mortality are local and regional recurrences, where the survival rate is about 50% in affected cases (9). Survival in oral cancer can be predicted using the usual variables such as tumor grade and depth of invasion. However, many biomarkers have been suggested as potential prognostic indicators of OSCC (10).

*KRAS* belongs to a group of GTP-binding proteins known as the Ras superfamily and also known as Ras-like GTPases. One hundred and fifty *Ras*-like genes or more have been identified in mammalians (11). *KRAS* protein is the *KRAS* gene product that contains 188 amino acid residues with a molecular weight of 21.6 kD and is considered one of the top-notch sensors that induce the activation of many signaling molecules in order to transmit the signals from the cell surface to the nucleus, thereby affecting the cellular growth, differentiation, apoptosis, and chemotaxis (12).

The* KRAS* gene mutation results in the appearance of oncogenic properties that are causally involved in the development of several types of cancers (13), including pancreatic carcinomas, liver cancer, colorectal cancer, lung carcinomas, biliary tract carcinomas, bladder cancer, endometrial cancer, cervical cancer, breast cancer, and myeloid leukemia (14-16). Few reports are available on the frequency of mutations in the *KRAS* gene in head and neck squamous cell carcinoma cases (17).

The present study aimed to examine the *KRAS* expression in oral epithelial dysplasia and squamous cell carcinoma of the oral cavity and to correlate its expression with the established prognostic markers** (**Ki-67, bcl2, and Cyclin D1) and the available clinicopathologic factors.

## Material and Methods

Forty paraffin-embedded OSCC excision biopsies with neck lymph node dissection were retrospectively collected. The age and gender of patients were retrieved from the clinical data in the histopathology request sheet form. Moreover, 20 paraffin blocks of oral leukoplakia with epithelial dysplasia cases were included. The specimens were collected from the Pathology Departments of two hospitals (Ahmed Maher teaching hospital and El Sahel teaching hospital) during the period from 2018 to 2021. The study was approved by the local Ethics Committee, Faculty of Medicine, Fayoum University.

**Histopathological Evaluation**

Four-μm thick re-cut was done from the paraffin blocks for each biopsy and stained with hematoxylin and eosin for routine histopathological examinations. The following histopathological features were evaluated: (a) confirmation of the diagnosis, (b) grading of squamous cell carcinoma according to the World Health Organization classification (4^th^ edition) (18), (c) assessment of the presence of lymph node metastases, perineural invasion (PNI), and lymphovascular invasion (LVI), and (d) pathologic staging according to American Joint Committee on Cancer (AJCC 8th edition) (19).

**Immunohistochemical Examination**

The 4-μm thick tissue sections were cut and mounted on slides. For specimens examined as conventional sections, de-waxing of the slides was followed by rehydration with sequential washes in ethanol and then in water. Antigen retrieval was done using sodium citrate buffer. Immunohistochemistry was performed using the rabbit polyclonal antibody against *KRAS* (orb53139 *KRAS* polyclonal antibody), rabbit monoclonal antibody to Cyclin D1 (SP4-R #790-4508), anti-Ki-67 rabbit monoclonal primary antibody (30-9 #790-4286), anti-Bcl-2 (SP66 #790-4604) rabbit monoclonal primary antibody, at a 1:50 dilution for 30 minutes, using the Vectastain Universal Elite ABC immunohistochemistry kit (with a 1:100 dilution of secondary antibody) and impact DAB peroxidase substrate detection reagent. Washed slides were then counter-stained in Gill’s hematoxylin. A secondary antibody (only for control) was included in each set of stained slides. All markers were used in OSCC sections, and only *KRAS* was used in addition to the stained sections of oral epithelial dysplasia.

**Immunohistochemical Scoring**

*KRAS* positivity was defined as brown cytoplasmic staining of tumor cells, and Cyclin D1 positivity was defined as brown cytoplasmic and nuclear staining of tumor cells. The scoring for *KRAS* and Cyclin D1 was calculated as follows: in each field, the total number of cells and the number of positive cells were counted, the average percentage of positive cells was detected, and the percentages were divided into four grades and recorded as 0, 1, 2, or 3. The used scoring system was: i) 0, <25% positive cells; ii) 1, 25 49% positive cells; iii) 2, 50 74% positive cells; and iv) 3, ≥75% positive cells (20).

Bcl2 positivity was defined as brown cytoplasmic staining of tumor cells, and Ki-67 positivity was defined as brown nuclear staining of tumor cells. The scoring for Bcl2 and Ki-67 was done according to the fraction of stained tumor cells: negative (0) when no positive cell was observed within the tumor, 1+ when up to 30% of the tumor cells were positive, 2+ when 31%–70% of the tumor cells were positive, and 3+ when 71–100% of tumor cells were positive (21).

For statistical purposes, negative and weakly positive expression in all markers was defined as low expression, while positive and strongly positive expression was defined as high expression.

**Statistical Analysis**

Microsoft Excel 2013 was used for data entry and the statistical package for social sciences (SPSS) version 22 (SPSS, Armonk, New York: International Business Machines Corporation) was used for data analysis. Simple descriptive statistics were used for qualitative data. The bivariate relationship was displayed in cross-tabulations, and a comparison of proportions was performed using the Chi-square test or Fisher’s exact test whenever appropriate. The independent samples t-test was used to compare normally distributed quantitative data. Pearson’s correlation coefficient was also used to compare normally distributed quantitative data. The level of significance was set at a probability of P-value ≤0.05.

## Results

**Fig. 1:**
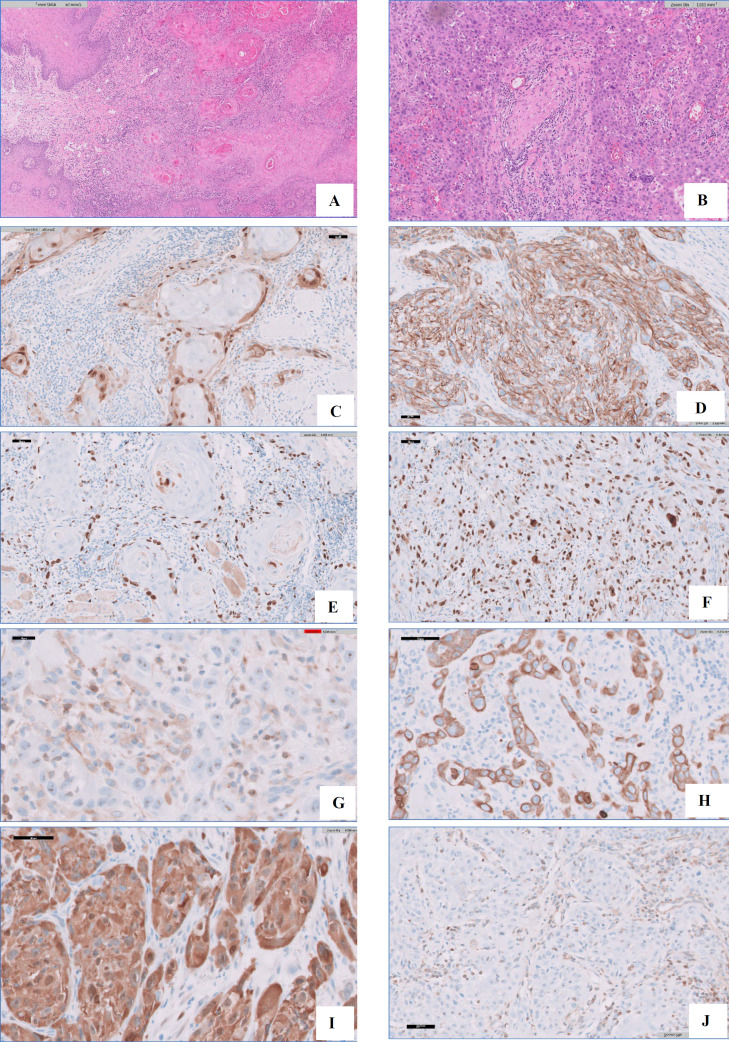
Oral Squamous cell carcinoma histological grade 1 (A) and grade 3 showing focal perineural invasion (B) (Hematoxylin and eosin X10) and immunohistochemical expression of *KRAS*, Ki-67, *bcl2,* and CyclinD1 in representative oral squamous cell carcinoma sections (X40). (C) low and (D) high expression of *KRAS*. (E) low and (F) high expression of Ki-67. (G) low and (H) high expression of Bcl2. (I) high and (J) low expression of CyclinD1

A total of forty cases diagnosed as OSCC (Figures 1A and 1B) were included in this study, along with 20 cases of oral leukoplakia with epithelial dysplasia (14 of them were diagnosed as mild epithelial dysplasia, 1 case was moderate, and 5 cases showed severe epithelial dysplasia).

The age of OSCC patients ranged from 30 to 90 years, with a mean of 63±13. The most frequent tumor site was the lips representing 45% of all cases. Only 5 patients (12.5%) showed distant metastasis. Detailed clinicopathological features of the studied OSCC patients and staining characteristics of the used markers are shown in Table 1.

**Table 1:** Clinicopathological features of the studied OSCC patients and staining characters of the used markers

**Variable**		**Number of cases (%)**
Age(y) mean ±SD		63±13
**Sex**	Male	29 (72.5)
	Female	11(27.5)
**Tumor site**	Tongue	13(32.5)
	Lip	18(45)
	Buccal mucosa	5(12.5)
	Gingiva	4(10)
**Histological grade**	Grade 1	19(47.5)
	Grade 2	1(2.5)
	Grade 3	20(50)
**Tumor extent** (according to TNM)	T0	1(2.5)
	T1	16(40)
	T2	6(15)
	T3	8(20)
	T4	9(22.5)
**Nodal status** (according to TNM)	N0	25(62.5)
	N1	5(12.5)
	N2	4(10)
	N3	6(15)
**Metastasis**	M0	35(87.5)
	M1	5(12.5)
**Pathological stage** (according to AJCC)	Stage I	14(35)
	Stage II	4(10)
	Stage III	7(17.5)
	Stage IV	15(37.5)
**Lymphovascular invasion**	Absent	25(62.5)
	Present	15(37.5)
**Perineural invasion**	Absent	23(57.5)
	Present	17(42.5)
***KRAS***	Low expression	23 (57.5)
	High expression	17 (42.5)
**Ki-67**	Low expression	16(40)
	High expression	24(60)
**Bcl2**	Low expression	17(42.5)
	High expression	23(57.5)
**Cyclin D1**	Low expression	18(45)
	High expression	22(55)

***KRAS*** **, Ki-67, Bcl2, and Cyclin D1 Staining Results and Their Association with Clinicopathologic Features of OSCC Cases**

*KRAS* was observed as tan or brown staining located mainly in the cytoplasm and in nuclei in only some regions. It was highly expressed in 17 (42.5%) cases and showed low expression in 23 (57.5) of the 40 studied OSCC cases. High *KRAS* expression was significantly associated with poorly differentiated OSCC (grade 3; *P*<0.001), higher tumor extent (*P*=0.001), the presence of nodal (*P*≤0.001) and distant metastasis (*P*=0.009), higher pathological stage (*P*<0.001), and the presence of lymphovascular invasion (*P*<0.001).

Ki-67 was expressed in all cases of OSCC, though with different scores. In well differentiated OSCC (n=19), the expression was located mainly at the periphery of the tumor nests than in the center, and most of these cases (n=15; 78.9%) showed low Ki-67 expression, while all poorly differentiated OSCC cases (n=20) showed high Ki-67 expression that was detected at the periphery of and at the tumor nests. Ki-67 expression was significantly associated with histological tumor grade (*P*<0.001), tumor extent (*P*=0.001), nodal metastasis (*P*=0.001), pathological stage (*P*<0.001), and the presence of lymphovascular invasion (*P*<0.001).

Bcl2 cytoplasmic expression was high in 32 (57.5%) and was low in 17 (42%) of OSCC cases. *Bcl2* expression showed significant relationships with histological grading (*P*<0.001), tumor extent (*P*=0.003), nodal metastasis (*P*=0.004), pathological stage (*P*<0.001), and the presence of lymphovascular invasion (*P*<0.001).

Cyclin D1 was mainly indicated as tan or brown nuclear and cytoplasmic staining. It showed high expression in 22 (55%) and low expression in 18 (45%) of the OSCC cases. This expression was significantly related to the tumor histological grade, tumor extent, nodal status, distant metastasis, lymphovascular invasion, and pathological stage (*P*=<0.001, <0.001, <0.001, 0.053, <0.001, and <0.001, respectively; Figure 1 and Table 2).

**Table 2:** The *KRAS* and other used markers expression in relation to clinicopathological parameters in OSCC

**Variable**		**Immunohistochemical expression **(total=40)											
		*KRAS*		*P*	Ki-67		*P*	Bcl2		*P*	Cyclin D1		*P*
		low	high		low	High		low	high		low	high	
Sex	Male	17	12	1	12	17	1	11	18	0.477	13	16	1
	Female	6	5		4	7		6	5		5	6	
Tumor site	Tongue	9	4	0.374	6	7	0.872	7	6	0.730	8	5	0.472
	Lip	8	10		6	12		7	11		6	12	
	Buccal mucosa	4	1		2	3		2	3		2	3	
	Gingiva	2	2		2	2		1	3		2	2	
Histological grade	Grade 1	18	1	<0.001*	15	4	<0.001*	15	4	<0.001*	17	2	<0.001*
	Grade 2	1	0		1	0		0	1		0	1	
	Grade 3	4	16		0	20		2	18		1	19	
Tumor extent (according to TNM)	T0	1	0	0.001*	1	0	0.001*	1	0	0.003	1	0	<0.001*
	T1	14	2		12	4		12	4		13	3	
	T2	4	2		2	4		2	4		3	3	
	T3	4	4		1	7		2	6		1	7	
	T4	0	9		0	9		0	9		0	9	
Nodal status (according to TNM)	N0	20	5	<0.001*	16	9	0.001*	16	9	0.004*	18	7	<0.001*
	N1	3	2		0	5		1	4		0	5	
	N2	0	4		0	4		0	4		0	4	
	N3	0	6		0	6		0	6		0	6	
Metastasis	M0	23	12	0.009*	16	19	0.071	17	18	0.061	18	17	0.053*
	M1	0	5		0	5		0	5		0	5	
Pathological stage (according to AJCC)	Stage I	14	0	<0.001*	13	1	<0.001*	13	1	<0.001*	14	0	<0.001*
	Stage II	4	0		2	2		2	2		3	1	
	Stage III	5	2		1	6		2	5		1	6	
	Stage IV	0	15		0	15		0	15		0	15	
Lymphovascular invasion	Absent	20	5	<0.001*	16	9	<0.001*	16	9	<0.001*	18	7	<0.001*
	Present	3	12		0	15		1	14		0	15	
Perineural invasion	Absent	13	10	0.884	10	13	0.601	10	13	0.884	11	12	0.676
	Present	10	7		6	11		7	10		7	10	

**Correlation between** ***KRAS*** **Expression and Clinicopathologic Features and the Expression of other Used Markers**

**Fig. 2:**
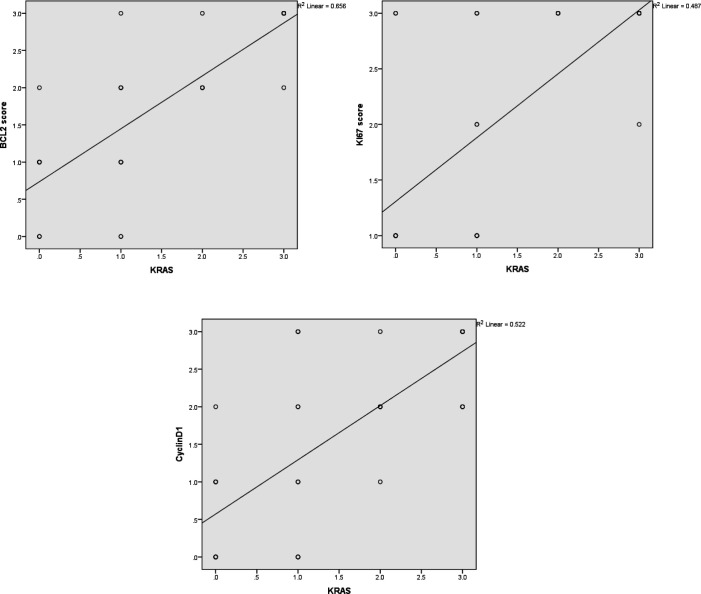
Correlation between *KRAS* expression and other used immunohistochemical markers

*KRAS* expression was positively correlated with several clinical and pathological indicators of the tumor progression, including the histological grade, the tumor extent, the nodal status, and the pathological stage (r=0.712, 0.649, 0.646, and 0.865, respectively; Table 3). It also showed a strong positive correlation with the expression of Bcl2 and Cyclin D1 (r=0.81 and 0.723, respectively) and a moderately strong positive correlation with Ki-67 expression (r=0.698; Figure 2).

***KRAS*** **Expression in Oral Leukoplakia with Epithelial Dysplasia**

**Fig. 3:**
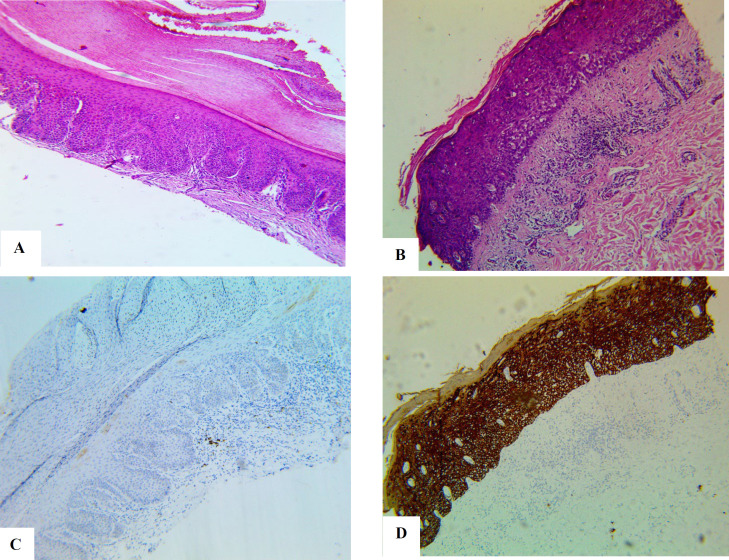
Oral leukoplakia with epithelial dysplasia (A) low grade dysplasia and (B) high grade dysplasia (Hematoxylin and eosin X10) and their representative immunohistochemical expression of *KRAS* (X10); (C) negative expression and (D) high expression

**Fig. 4:**
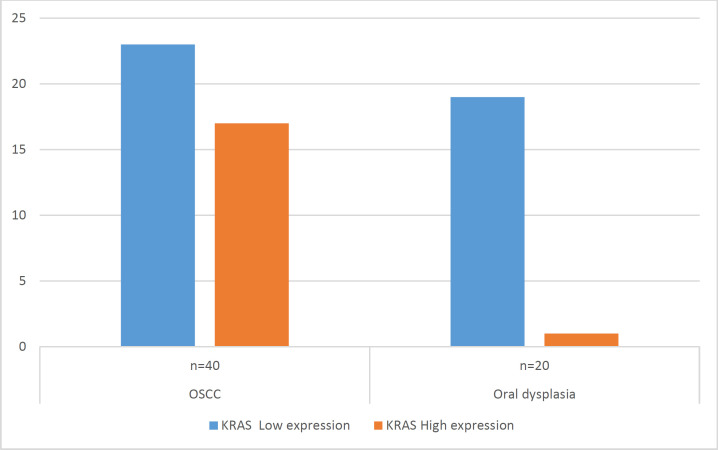
Expression of *KRAS* in OSCC and in oral leukoplakia with epithelial dysplasia

On the immunohistochemical evaluation of *KRAS* staining, only one case (5%) of the 20 examined cases of oral epithelial dysplasia showed high expression, while the rest of the cases showed no or weakly positive expression (low expression; Figure 3). This expression was significantly lower than the *KRAS* expression in OSCC (*P*=0.003; Figure 4).

**Table 3:** Correlation between *KRAS* expression and clinicopathologic features and the expression of other used markers:

**Variable**		***KRAS*** ** expression**
Histological grade	R-value	0.712
	P-value	<0.001*
Tumor extent (according to TNM)	R-value	0.649
	P-value	<0.001*
Nodal status (according to TNM)	R-value	0.646
	P-value	<0.001*
Pathological stage (according to AJCC)	R-value	0.865
	P-value	<0.001*
Ki-67 expression	R-value	0.698
	P-value	<0.001*
CyclinD1 expression	R-value	0.723
	P-value	<0.001*
*bcl2* expression	R-value	0.810
	P-value	<0.001*

## Discussion

*KRAS* mutations are considered the most common type of Ras mutations in all types of human cancers (22). Although *KRAS* gene mutations are infrequent in OSCC (23), several studies have shown that miRNA binding site polymorphisms in *KRAS* were associated with reduced survival time in oral cancer (24,25). 

The *KRAS* is one of the most reliable predictors of resistance to targeted therapy using EGFR1 tyrosine kinase inhibitors (26). There has been a significant improvement and advancement in the field of targeted therapy that resulted in the successful targeting of *KRAS* or downstream effects in many types of cancers in clinical trials. However, a proper understanding of the mechanisms associated with *KRAS* activity in cancer will help in identifying proper therapeutic strategies (27).

OSCC is a malignant epithelial neoplasm of the oral mucosa that has multifactorial pathogenesis (28). Histologically, the process passes through many phases of potentially malignant changes prior to the establishment of invasive carcinoma. These preneoplastic changes include mild, moderate, and severe epithelial dysplasia (29).

A recent study by Wang et al. referred to the role of *KRAS* in OSCC filtered the genes relevant to OSCC and performed 29 cases of immunohistochemical staining at different cellular portions. The authors combined these stains into pairs and identified novel immunohistochemical prognostic markers in OSCC, including *KRAS*, among the Taiwanese population (30).

Recently, *KRAS* has been prominently noticed in the field of head and neck cancer research due to its strong relationship with tumorigenesis, invasion, lymph node metastasis, recurrence, and prognosis (20). The current study showed that *KRAS* expression was significantly higher in OSCC compared to oral leukoplakia with epithelial dysplasia. Its expression was also significantly higher in poorly differentiated OSCC than in well-differentiated tumors. This may refer to a role played by *KRAS* in the progression of OSCC.

In addition, in this study, several clinical and pathological indicators of the tumor progression, such as tumor extent, nodal metastasis, distant metastasis, pathological stage, and lymphovascular invasion, have been related to *KRAS* expression, indicating that this protein expression may be closely associated with occurrence and development of OSCC, and it may be hypothesized that increased *KRAS* expression promotes OSCC invasion and metastasis.

Recently, much attention has been paid to a particular panel of molecular markers, which includes proliferative and cell cycle regulatory molecules as possible predictors of the biological behavior of OSCC (31). Thus, the current study examined the expression of a number of these markers. 

Ki-67 is one of the most commonly used tumor markers for studying cellular proliferation, and its expression in OSCC was examined in many previous studies that showed similar results to that of the current study, as high Ki-67 expression was mostly associated with adverse clinicopathological parameters such as histological tumor grade, tumor extent, nodal metastasis, TNM stage, and lymphovascular invasion (32,33).

*Bcl2* is a protooncogene that regulates programmed cell death (apoptosis). It contributes to the neoplastic process by prolonging the neoplastic cell survival by inhibiting programmed cell death (34). It is also well studied in OSCC in several previous studies that reported Bcl2 expression correlated with nodal metastasis and TNM stage (35). Its low expression is usually related to better OSCC differentiation and also a good prognosis (36).

The accumulation of alterations in genetic and epigenetic oncogenes and tumor suppressor genes, as in other cancers, may lead to the formation of OSCC. One of the genes that are obviously altered in OSCC includes *Cyclin D1* (37). Cyclin D1 protein plays a major role in cell cycle regulations as it controls the transition from G1 to S phase and thus regulates the rate of cellular proliferation (38). In addition, cyclin D1 overexpression has an important role in cooperating with other protooncogenes in the neoplastic process (39), and its overexpression was an important genetic event in OSCC (40).

Cyclin D1 was associated with tumorigenesis and proliferation of oral cancer (41). Several studies showed that cyclin D1 expression was associated with decreased patient survival and poor prognosis in OSCC (42).

In our study, Cyclin D1 high expression was significantly associated with the well-differentiated tumor, advanced tumor extent, nodal and distant metastasis, lymphovascular invasion, and pathological stage. This mostly goes with the results of the previous studies (43,44).

## Conclusion

One limitation of this study was the relatively small number of studied cases. However, *KRAS* expression was positively correlated with the expression of Ki-67, *bcl2*, and Cyclin D1, and all of them were closely associated with important clinicopathological prognostic markers in OSCC patients. In addition, a significant increase was found in *KRAS* expression in OSCC rather than in premalignant oral epithelial dysplasia. Hence, the present study attempted to highlight the possible role of *KRAS* expression in the progression and development of OSCC and its probable prognostic value. Further studies on a bigger number of cases are highly recommended.

## Ethics Approval

The study was approved by the Institutional Medical Ethical Committee (Faculty of Medicine, Fayoum University) (Mentioned inside the text in the method section) **and consent to participate: **Not applicable

## Authors' Contributions

 The manuscript has been read and approved by all the authors; HMEH: Conception and design; HMEH and MAAS: Analysis of data, Interpretation of data

## Conflict of Interest

The authors declared no conflict of interest.

## Funding

The authors would like to confirm that they covered the expenses of this research work completely on their own and they were not funded by any institution in Egypt.
